# Employment in Later Life: Its Multiple Benefits, Challenges, and Future Outlook

**DOI:** 10.1111/ggi.70629

**Published:** 2026-07-26

**Authors:** Yoshinori Fujiwara, Hiroshi Murayama

**Affiliations:** ^1^ Tokyo Metropolitan Institute for Geriatrics and Gerontology Tokyo Japan; ^2^ Research Team for Social Participation and Healthy Aging Tokyo Metropolitan Institute for Geriatrics and Gerontology Tokyo Japan

**Keywords:** employment, frailty, multiple benefits, older worker

## Abstract

Amid rapid population aging, declining fertility, and a shrinking workforce, employment in later life has become a critical issue in Japan. Older adults are no longer viewed solely as care recipients; many remain engaged in paid work, making employment a key form of social participation. This review synthesizes epidemiological evidence on the health benefits, challenges, and future directions of late‐life employment, drawing on the authors' previous studies and domestic and international cohort research from a health and welfare policy perspective. Longitudinal evidence indicates that continued employment is associated with reduced mortality, better self‐rated health, preserved cognitive function, and maintained activities of daily living. However, these benefits vary by sex, employment type, and frailty status. Notably, full‐time employment may offer protective effects even among frail older adults, particularly in reducing risks related to dementia and physical decline, whereas irregular or sporadic work provides limited benefits. The motivation and meaning of work also play a crucial role. Employment driven solely by financial necessity is linked to poorer health outcomes and greater social isolation, while nonfinancial motivations—such as maintaining health, purpose in life, and social contribution—are associated with more favorable outcomes. Finally, the study underscores the importance of community‐based intermediaries and emerging approaches such as social prescribing to connect older adults with meaningful, ability‐appropriate work. Effective support requires integrating employment assistance with frailty assessment, safety management, and multidisciplinary coordination.

## Graphical abstract

Employment in later life promotes healthy aging by reducing mortality, preserving cognitive and functional capacity, and enhancing social participation. Its benefits depend on work type, frailty, and motivation, highlighting the importance of meaningful, ability‐appropriate work supported by community‐based coordination and social prescribing.

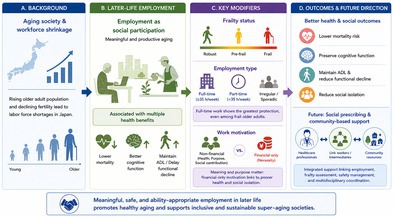

## 1 Introduction

In recent years, an increasing number of older adults have continued to engage in paid employment. The labor force participation rate among the older adult population has steadily increased, reflecting broader demographic trends of population aging and workforce shrinkage. According to the 2023 Labor Force Survey by the Statistics Bureau of Japan, labor force participation rates were 53.5% for those aged 65–69 years, 34.5% for those aged 70–74 years, and 11.5% among individuals aged ≥ 75 [1]. These figures challenge traditional stereotypes of older adults as dependent on long‐term care or having ample free time. In clinical practice, older patients should be regarded similarly to working‐age adults, with attention to their active engagement in society.

In the context of low fertility, super‐aging populations, and overall population decline, older adult employment is increasingly viewed as a critical strategy to supplement the shrinking labor force and potentially transform older adults from social security recipients into active taxpayers. The Japanese government has actively promoted employment continuation through measures such as the revision of the Act on the Stabilization of Employment of Older Persons, effective April 1, 2021, which encourages extending the mandatory retirement age to 70 [2]. Beyond its economic and labor‐related significance, employment later in life has important positive implications for individual health and social participation. It contributes to the maintenance of physical and mental health, provides opportunities for social engagement, and supports cognitive and functional capacities.

Madero‐Cabib et al. examined how the relationship between later‐life employment and health varies across different welfare state contexts using a harmonized pooled‐country data set of 12 nations [3]. Their findings showed that the association between later‐life employment trajectories and health varies across welfare state regimes. In more protective institutional contexts, continued employment is more likely to reflect positive health selection, resulting in neutral or beneficial health outcomes. In contrast, in less protective contexts, continued employment may include individuals in poorer health, leading to less favorable health associations. According to the Cabinet Office's 2022 White Paper on the Aging Society [4], 51.6% of adults aged ≥ 65 years participated in “social activities,” indicating that more than half of this population is socially engaged. Participation was highest in “health and sports” activities (27.7%), followed by “hobby” activities (14.8%). These figures reflect engagement over 1 year and allow for multiple responses. Furthermore, a survey conducted by the Consumer Affairs Agency in the same fiscal year reported participation rates in volunteer or community service activities of 16.4% among individuals aged 65–74 years and 15.7% among those aged ≥ 75 years [5]. According to the same White Paper, 30.2% of older adults were engaged in paid employment, indicating that employment remains relatively high compared to those participating in other social activities. Among currently employed individuals aged ≥ 60 years, approximately 40% expressed a desire to continue working as long as they were physically able. Considering the high labor force participation rate, paid employment remains the primary form of social engagement among older adults. These findings provide critical insights into the patterns of social participation in later life and inform policy design for older adults. Reflecting on this perspective, municipalities increasingly promote older adult employment not only through economic development departments but also through community‐based care divisions focusing on preventive care, frailty prevention, and the promotion of social engagement.

The aim of this article was to examine the effects of employment in later life on health outcomes, primarily drawing on the author's previous research while also referring to evidence from domestic and international cohort studies. In addition, it seeks to discuss the challenges and future prospects surrounding employment among older adults, situating the analysis within a broader context, from the perspective of health and welfare policies for the aging population in Japan.

## 2 Employment in Later Life and Health

Productive activity is defined as any activity that generates social value, regardless of whether it is paid or unpaid [6]. Unpaid activities include volunteer work, whereas paid activities comprise both employment‐based paid work under formal contracts and nonemployment‐based paid activities without such contracts. In this study, “Employment in Later Life” includes both employment‐based and nonemployment‐based paid activities of older adults aged ≥ 60 years.

A substantial body of domestic and international research has investigated the effects of employment on physical and mental health in later life. Recent systematic reviews of longitudinal studies have demonstrated that continued engagement in paid work is generally associated with reduced mortality risk and better overall health. In one review of 37 832 records, 14 studies met the inclusion criteria. Eight studies were conducted in Asia (four in Japan, two in Taiwan, and one each in Thailand and South Korea), three in the United States, two in Israel, and one in Brazil. Of 14 studies, 13 studies reported a positive association between late‐life employment and reduced mortality risk [7]. Regarding overall health measured by self‐rated health, five studies were included (four from Japan and one from the United States). Three of these studies demonstrated that employment later in life can suppress health deterioration [8]. Similarly, a systematic review examining cognitive function in older workers found that five of six prospective studies reported a protective effect of employment against cognitive decline. Sex‐specific analyses of three studies indicated heterogeneous results, suggesting that the impact of employment on cognitive function may differ between men and women [9]. Overall, these findings indicate a consistent positive effect of late‐life employment on physical and cognitive health.

In a region‐specific cohort study conducted by Fujiwara et al., men who remained employed, regardless of rural or urban residence, demonstrated a considerably lower decline in basic activities of daily living over an 8‐year follow‐up [10]. Notably, labor force surveys indicate that older men continue to engage in employment at rates approximately 20% higher than those of older women, suggesting that continued employment may play a significant role in maintaining functional capacity among older men, who are generally considered less inclined to participate in social activities than their female counterparts. However, the health benefits of employment are not uniform across sexes. A cross‐sectional study of Japanese adults aged ≥ 60 years revealed that male participants' walking activity, sedentary behavior, and sense of self‐usefulness varied significantly according to employment status (e.g., reemployment or retirement), whereas such differences were not observed among women [11]. Another study analyzing employment patterns among adults aged 60–85 years found clear sex differences in labor participation contingent on employment type (wage labor vs. self‐employment), socioeconomic status, and family structure. Employment patterns were strongly influenced by family responsibilities, demonstrating the interplay between work, gender roles, and cultural norms [12]. These findings suggest that men's employment is more strongly linked to physical activity and self‐perceived usefulness, whereas women's employment may be influenced by family roles and previous work history. Further research, including detailed analysis of employment type, health outcomes, and time allocation, is required to elucidate these sex‐specific patterns. Expanding both quantitative and qualitative data on the employment of older women, including regular, nonregular, and reemployment forms, is essential given their increased workforce participation [13].

Thus, previous studies have generally concluded that employment is associated with the prevention of health decline among older adults. However, a major challenge in social epidemiology is reverse causality, whereby healthier individuals are more likely to remain employed, leading to potential selection bias. Although the cited longitudinal studies adjusted for baseline health status, they remain observational in nature rather than interventional and thus cannot fully eliminate the possibility of the “healthy worker effect.” In randomized controlled trials, direct allocation of employment status (i.e., employment vs. nonemployment) among older adults is rarely feasible due to ethical and practical constraints, as individuals cannot be randomly assigned to work or not work against their will. Therefore, as a second‐best approach, it is desirable to conduct longitudinal analyses that explicitly stratify participants by baseline health status and rigorously adjust for potential confounding factors.

## 3 Employment Benefits for Frail Older Adults

Employment in later life is often assumed to be limited to relatively healthy older adults, with benefits restricted to this subgroup. To address this, we conducted a longitudinal analysis examining the effects of older adults' employment status and frailty on the risk of care‐need certification, stratified by primary cause [14].

**FIGURE 1:**
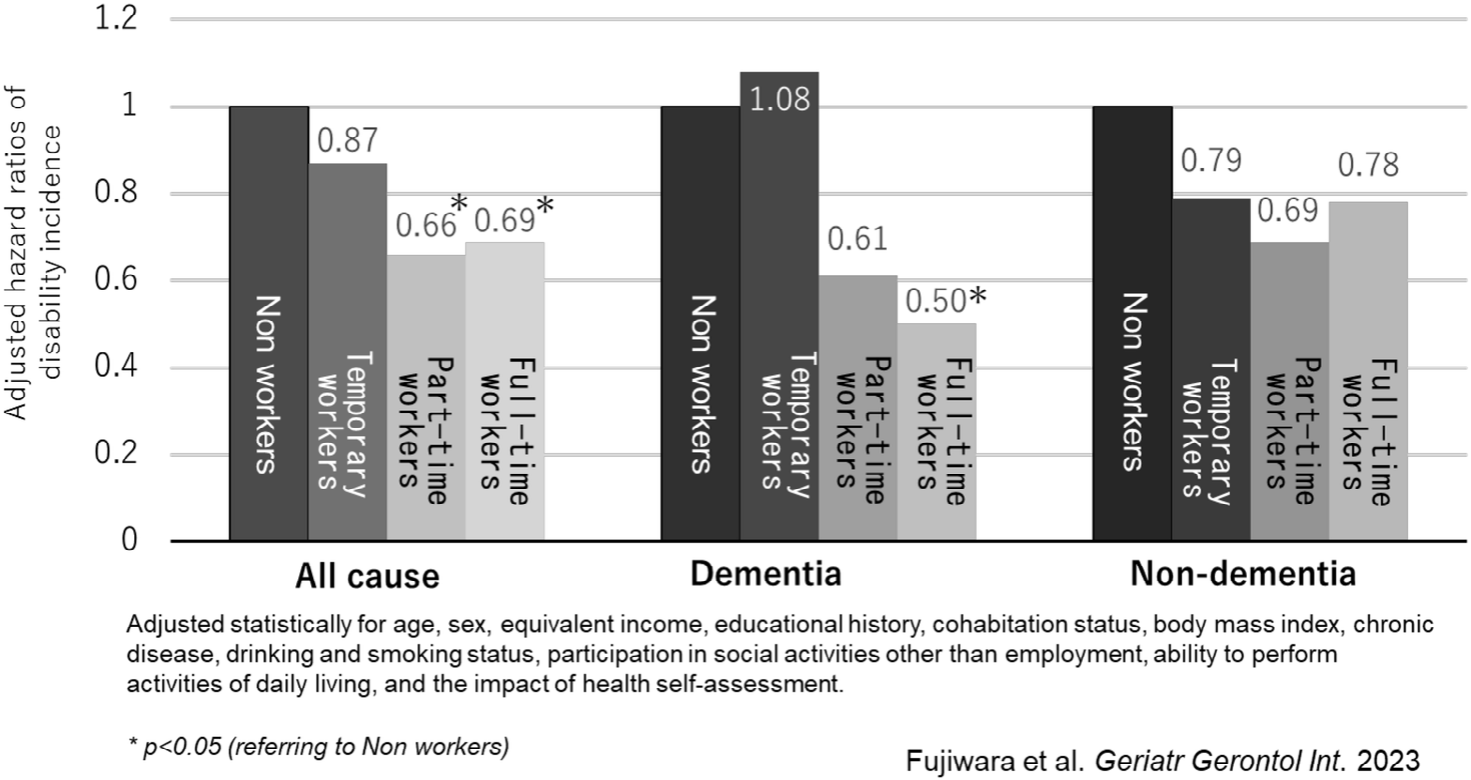
Adjusted hazard ratios of disability incidence for working status without frailty by Cox proportional models.

**FIGURE 2:**
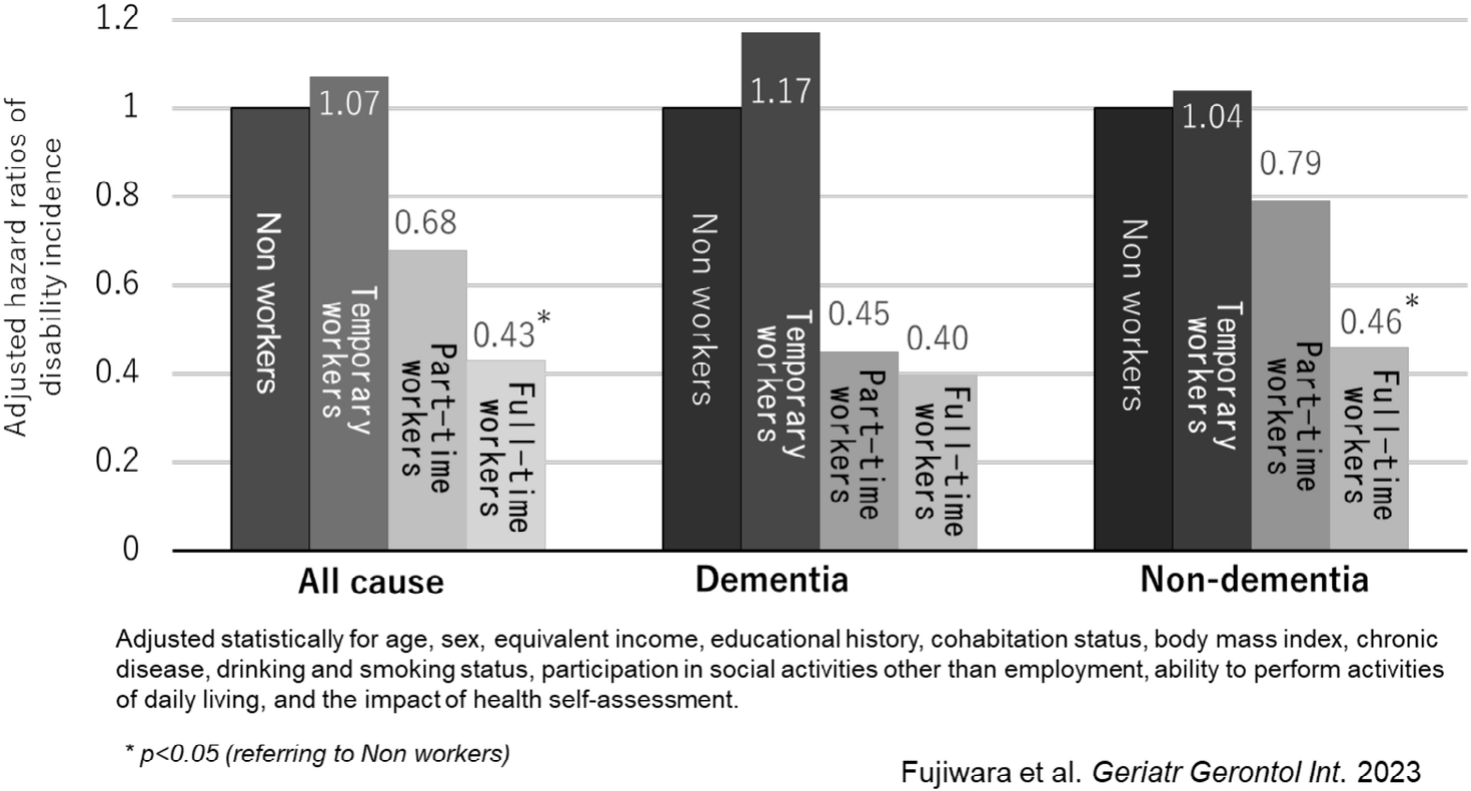
Adjusted hazard ratios of disability incidence for working status with frailty by Cox proportional models.

In a prospective study in Tokyo, Japan, 6386 men and women aged 65–84 years were followed up for 3.6 years to examine the interaction between frailty status, employment type, and subsequent risk of new certification for long‐term care needs [14]. Employment status at baseline was categorized as nonemployed (*n* = 3704), full‐time (≥ 35 h/week, *n* = 1134), part‐time (< 35 h/week, *n* = 1001), and irregular (*n* = 547). Among full‐time and part‐time workers, 17.5% and 15.3%, respectively, were classified as frail. Outcomes were analyzed according to the primary cause of long‐term care certification, distinguishing between dementia‐ and non‐dementia‐type disabilities, the latter primarily due to physical function decline [14]. Over the follow‐up, 806 participants (12.6%) were newly certified to receive long‐term care. Incidence rates varied by employment status: unemployed (16.8%); full‐time (5.6%); part‐time (5.8%); and irregular (11.2%). Figure 1 illustrates that both full‐time and part‐time employment reduced the overall risk of new care certification by 31%–34% compared to their nonemployed peers among robust individuals. In contrast, as shown in Figure 2, among frail participants, only full‐time employment significantly reduced the risk by 57%, indicating that structured, consistent work may be necessary to confer measurable health protection [14]. When disaggregated by cause, full‐time employment among robust older adults was associated with a 50% reduction in dementia‐related care certification, whereas frail individuals experienced a 54% reduction in non‐dementia‐related certification. These findings underscore that full‐time employment may facilitate cognitive maintenance in robust older adults and preserve physical function in frail older adults. Irregular or sporadic employment appeared insufficient to produce protective effects, likely because of inadequate cumulative engagement in social and physical activities.

Among robust older adults, both full‐time and part‐time employment have been found to reduce the overall risk of new care‐need certification. In frail older adults, full‐time employment may substantially lower the risk of new certification. Although part‐time work in frail individuals tended to reduce the risk, the difference was not statistically significant. Irregular or sporadic employment alone was not associated with a considerable preventive effect. In the robust group, many individuals engaged in additional social activities, such as hobbies, sports, or volunteering, whereas frail individuals were less likely to participate in social activities outside of work. Given that higher total engagement in social activities, including employment, has been associated with care‐prevention benefits, part‐time work alone may not provide a sufficient overall level of social activity for frail older adults [14]. Accordingly, compared with robust older workers, frail older workers—particularly those engaged in part‐time employment—may have fewer opportunities to proactively devote their nonworking time to other forms of social participation, such as leisure activities or volunteering. From this perspective, it can be hypothesized that, for frail older adults, full‐time employment may be more advantageous than part‐time work in maintaining an adequate overall level of social activity in daily life. However, it is also true that, for a frail individual, full‐time work could plausibly lead to exhaustion or an increased risk of injury. Therefore, it is important to examine strategies that would enable frail older adults to engage in full‐time work in a safe and sustainable manner.

To this end, full‐time employment among frail older adults is feasible under job designs with low physical demands, high flexibility, and decentralized tasks. Job design, rather than occupational type alone, appears decisive for sustained employment. These findings are consistent with the job demand–control model, where high demands and low control increase strain and health risks [15]. Frail older adults are particularly sensitive to high demands, while low‐demand, high‐control jobs support sustainable work. Similarly, the Job Demands–Resources model highlights that autonomy, flexibility, and social support buffer the effects of demands [16]. Decentralized task allocation enhances person–job fit and allows adaptive adjustment to individual capacity. Job crafting, or proactive modification of tasks by workers, can further improve alignment between capabilities and job requirements, enabling continued employment despite functional limitations [17].

In summary, reducing demands, increasing resources, and decentralizing tasks collectively support full‐time work among frail older adults. These findings emphasize the importance of job design–focused strategies in aging workforce policies. Future research should use longitudinal designs and objective measures to confirm these deductions. To promote desirable employment among frail older adults, it is essential to carefully examine the types of tasks and work arrangements that can be performed on a full‐time basis despite frailty and to disseminate this information to employers and those supporting older adults in employment.

## 4 Motivation and Meaning of Work for Older Adults

Although employment confers measurable health benefits, the purpose and meaning of work critically determine its effectiveness. Evidence from large‐scale longitudinal studies in the United States and Europe suggests that motivations for labor force participation in later life are shaped by economic, health‐related, and institutional factors. The Health and Retirement Study shows that retirement is often not a permanent exit from the labor market, as many older adults return to work. Key motivations include financial necessity, such as insufficient retirement savings or unexpected financial shocks, as well as nonfinancial factors such as job satisfaction and intrinsic motivation to remain active [18]. In Europe, research based on the Survey of Health, Ageing and Retirement in Europe indicates that retirement behavior is strongly influenced by national welfare systems in addition to individual factors such as health status and job quality. Retirement tends to be more voluntary in Northern and Western Europe, whereas financial necessity plays a greater role in Southern Europe [19].

**FIGURE 3:**
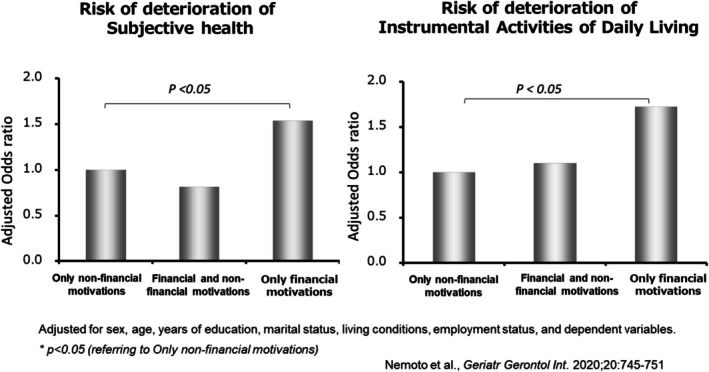
Employment motivation in old age and risk of health deterioration after 2 years.

Overall, later‐life work decisions reflect a multidimensional interaction of economic, health, and institutional contexts. Not all work engagement is equally beneficial; the underlying motivation of older workers, whether for financial necessity or personal fulfillment, influences psychological and physical health outcomes, as well as social integration [20]. In a postal survey of 7608 community‐dwelling older adults aged ≥ 65 years in Tokyo [20], 1069 employed participants at baseline were analyzed. Employment motivations were classified into two categories: nonfinancial motives, including health maintenance, finding purpose in life, and making social contributions; and financial motives, including earning a living, repaying debt, or generating supplemental income. Participants were further stratified into three groups: nonfinancial, combined nonfinancial and financial, and financial only. Figure 3 shows employment motivation in old age and the risk of health deterioration. A longitudinal analysis over 2 years revealed striking differences in health outcomes based on motivation. Participants working solely for financial reasons exhibited a 1.42‐fold higher risk of deterioration in self‐rated health and a 1.55‐fold higher risk of functional decline than those with nonfinancial motivations [20]. In contrast, individuals with combined motivations experienced health outcomes comparable to those working exclusively for nonfinancial reasons, suggesting that the presence of eudaimonic incentives mitigates potential stressors associated with financial necessity [20].

Economic motivation for work, which is often regarded as a proxy for low socioeconomic status, is an independent risk factor for poorer health outcomes. Considering the potentially confounding influence of socioeconomic status on work motivation, it is necessary to carefully examine whether the observed adverse effects are attributable to the economic motivation itself or to the nature of the work typically undertaken by individuals driven by necessity, which is often characterized by low wages and high demands. In this study, household income and employment status at baseline were adjusted for; however, such adjustments may not fully account for these confounding factors. Nevertheless, it is possible to complement economic motivation with other forms of motivation.

These findings align with theoretical models of occupational engagement that emphasize meaningful participation as a driver of well‐being. Work that provides a sense of purpose, social connectedness, or perceived contribution to society offers psychosocial stimulation, reinforces social networks, and reduces the risk of social isolation and associated negative health outcomes [20]. In light of Maslow's hierarchy of needs, work motivation in later life should not be reduced to economic necessity alone, even among financially constrained older adults [21]. Employment may also fulfill higher‐order needs, including belongingness, esteem needs, and self‐actualization. From this perspective, workplace practices—such as health‐oriented management, supportive interpersonal relationships, and opportunities to experience appreciation—can enhance the psychosocial value of work. Conversely, employment motivated solely by financial necessity may impose physical and psychological stress, particularly if work demands involve long hours, heavy labor, or hazardous conditions, which can exacerbate frailty or functional decline [20].

To examine the relationship between employment motivation and social isolation, the same cohort was assessed for social engagement over a 2.5‐year follow‐up. Social isolation was defined as engaging with non‐cohabiting family or friends less than once per week. Among employed individuals, those motivated solely by financial reasons demonstrated a 3.65‐fold higher risk of developing social isolation than those motivated by nonfinancial factors [22]. Participants with combined motivations exhibited intermediate risk, reinforcing the protective role of eudaimonic engagement against social disconnection.

The mechanisms underlying these observations likely involve both behavioral and psychological pathways. Nonfinancially motivated work encourages frequent interpersonal interactions, cooperative problem‐solving, and participation in team‐based activities, strengthening social networks and enhancing cognitive engagement [23]. In addition, perceiving one's work as meaningful reinforces self‐efficacy and usefulness, which are linked to improved mental health and a reduced risk of depressive symptoms. In contrast, financial‐motivation‐only employment may lack these psychosocial benefits, as work is primarily instrumental, task‐oriented, and potentially performed in isolation or under stressful conditions [23].

International literature corroborates these findings. Studies from Europe and North America indicate that older adults engaged in purpose‐driven work or volunteer activities exhibit better cognitive function, lower rates of depressive symptoms, and higher overall well‐being than those employed solely for economic reasons [24]. For instance, research in the United Kingdom demonstrated that older adults participating in paid or unpaid roles fostering skill utilization, social contribution, or creative engagement report superior mental health outcomes over 5 years [24]. Similarly, longitudinal analyses in the United States suggested that employment perceived as meaningful reduces the risk of functional decline even after adjusting for baseline health and socioeconomic factors [24]. These findings suggest that employment policies and workplace design for older adults should prioritize job quality, not merely labor force participation. Programs that incorporate flexible schedules, task variety, social interaction, and recognition can enhance both health and social outcomes [25]. In contrast, policies that overlook the nature and meaningfulness of work risk limited effectiveness and may widen health disparities among economically vulnerable groups [25]. Therefore, promoting holistic health through later‐life employment requires integrating economic and psychosocial aspects of work, supported by employer awareness, organizational commitment, and institutional frameworks that foster health‐conscious management and supportive workplace relationships.

## 5 Coordination and Community‐Based Work: Social Prescribing and Multidimensional Employment

**FIGURE 4:**
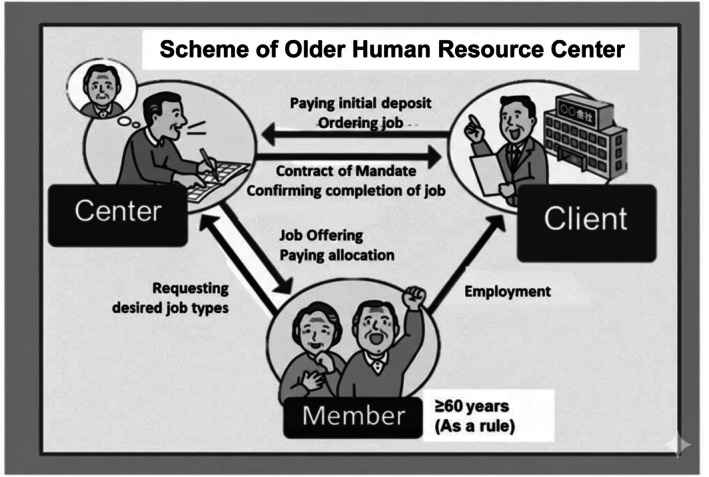
Scheme of the Older Human Resources Center, also known as the Silver Human Resources Center (SHRC).

Figure 4 below summarizes the scheme of the Older Human Resources Center, also known as the Silver Human Resources Center (SHRC). To promote employment among older adults, intermediaries are required to match and coordinate job‐seeking older individuals with employers, such as the SHRC [26] and municipality‐specific job‐matching initiatives [27]. SHRCs are community‐based nonprofit organizations in Japan that provide employment opportunities for older adults, generally aged ≥ 60 years. Established under the Act on the Stabilization of Employment of Older Persons, SHRCs aim to promote social participation, improve quality of life, and support active aging by enabling retirees to engage in temporary, short‐term, and light work.

Unlike conventional employment, work arrangements in SHRCs are typically based on a subcontracting system in which members undertake work on a contractual basis rather than a standard employment contract. Jobs mainly include community‐oriented services such as cleaning, park maintenance, clerical assistance, childcare support, and other locally needed services. Through these activities, SHRCs contribute to the well‐being of older adults and the sustainability and vitality of local communities [26].

A 2‐year longitudinal study demonstrated that participants working through the SHRC considerably increased their rate of pre‐frailty improvement [28]. In contrast, safety is a paramount concern in older worker employment, particularly in physically demanding or hazard‐prone environments, because the average age of the member is approximately 75 years [29]. National labor statistics indicate that the proportion of occupational injuries among workers aged ≥ 60 years is increasing, emphasizing the need for targeted risk mitigation strategies [29]. SHRCs exemplify structured approaches to safety, offering training, guidance, and placement support for older adults. Nevertheless, empirical data suggest that pre‐frail and frail individuals are at higher risk for accidents, including falls, slips, and object‐related incidents, and are less likely to use safety training opportunities [28]. Therefore, integrating frailty assessments and tailored safety interventions within employment programs is essential to optimize both productivity and health outcomes.

The Organization for Economic Co‐operation and Development indicates that older workers are more likely to experience chronic health conditions and fatigue and that workplace stress and high job demands may exacerbate their health problems [30]. In addition, the World Health Organization reports that due to age‐related functional decline, the same level of occupational workload can have a more pronounced adverse impact on the health of older adults [31]. These issues are common among older workers in general; however, for frail older adults with reduced physiological reserves, job design–focused strategies are particularly important, as noted above. Nevertheless, in reality, the relationship between work and health in later life is influenced by a wide range of factors involving workers themselves, employers, families, and society at large, meaning that ideal job design cannot always be achieved. In this context, an integrated healthcare approach by primary care physicians, which considers the working lives of older patients, is expected to play an important role in supporting healthy aging and continued employment.

It is also necessary to consider life after eventual retirement from employment. In the future, it will be necessary not only to focus on the prevention of frailty among healthy older adults but also to examine what types of work or productive activities enable individuals to maintain social roles, avoid social isolation, and continue working in ways consistent with their personal values even after becoming frail. Activities should be matched appropriately to individuals based on their capacities and circumstances. Such initiatives align conceptually with *social prescribing*, an approach that originated in the United Kingdom, whereby healthcare professionals offer nonmedical interventions to improve patients' health and well‐being [32], social prescribing involves matching individuals through coordinators, or link workers, to community‐based social activities, including hobby groups, agricultural work, and artistic or handcrafted activities. In Japan, there is growing interest in models where primary care physicians collaborate with community‐based coordinators for paid social activities, including employment, particularly for patients with frailty, mild cognitive impairment, or dementia [33].

In a highly competitive labor market, securing work that matches an individual's abilities is challenging. Japan lacks a formal framework to appoint link workers as statutory officers, as seen in the United Kingdom; therefore, it is necessary to define equivalent roles and frameworks within the Japanese context. In practice, medical social workers, community life support coordinators [34], community comprehensive support center staff, and care managers may partially fulfill such functions.

In addition, “Employment‐oriented Activity Support Coordinators,” [34] as described in national guidelines, play a key role in bridging the gap between welfare services and the labor market. The core aim of this support is to enable individuals to achieve desired social roles by facilitating participation in various activities, including paid work, nonemployed paid roles, and volunteering. Two key perspectives are essential: individualized matching based on a person's abilities and circumstances and the development of diverse opportunities that allow continued participation even as functional status changes. Thus, employment‐oriented activity support can be understood as a form of social participation support. In Japan, its function as a “link worker” substitute is achieved through collaboration between welfare‐sector coordinators and employment‐oriented activity support coordinators.

Challenges of social prescribing include the uneven distribution of community resources, the need to ensure the expertise of providers and appropriate incentives, the difficulty of defining outcome measures, and the establishment of effective information‐sharing systems between healthcare services and community organizations. Nevertheless, social prescribing is increasingly recognized as a promising approach that leverages community assets to address health needs that cannot be fully managed by medical care alone. Given this context, geriatricians are expected not only to support the health and safety management of robust older workers but also to provide appropriate social prescribing, including diverse forms of employment, for older adults at high biopsychosocial risk and to assume leadership in coordinating multidisciplinary support for them.

## 6 Conclusion

Employment in later life plays a crucial role in promoting healthy aging, going far beyond its economic relevance. Accumulating evidence indicates that continued engagement in paid work is associated with lower mortality risk, better self‐rated health conditions, preserved cognitive function, and delayed functional decline. Notably, these benefits are observed not only among robust older adults but also among those with frailty, particularly when employment is structured, continuous, and sufficiently engaging. These findings challenge the assumption that later‐life employment is suitable only for healthy individuals and also emphasize its potential contribution to long‐term care prevention. Simultaneously, the effects of employment are heterogeneous. Sex differences, employment type, work intensity, and especially work motivation substantially influence health and social outcomes. Employment motivated solely by financial necessity is associated with poorer health outcomes and a higher risk of social isolation, whereas work that provides meaning, purpose, or opportunities for social contribution confers psychosocial benefits and supports sustained well‐being. Thus, the quality and meaning of work are as important as participating in it.

Future strategies should adopt a person‐centered, integrated approach that links employment policy with public health, community care, and social prescribing. Coordinated systems that connect older adults to safe, meaningful, and capacity‐appropriate work, supported by intermediaries and healthcare professionals, may help extend healthy life expectancy and foster inclusive, age‐integrated societies in super‐aging contexts.

## Ethics Statement

The authors have nothing to report.

## Conflicts of Interest

The authors declare no conflicts of interest.

## Data Availability

The authors have nothing to report.

## References

[1] Statistics Bureau, Ministry of Internal Affairs and Communications , “Labor Force Survey: Annual Report on the Labor Force Survey 2023,” 2024, https://www.stat.go.jp/english/data/roudou/index.html.

[2] Ministry of Health, Labor and Welfare (Japan) , Act on Stabilization of Employment of Older Persons (Ministry of Health, Labor and Welfare, 2021).

[3] I. Madero‐Cabib , L. Corna , and I. Baumann , “Aging in Different Welfare Contexts: A Comparative Perspective on Later‐Life Employment and Health,” Journals of Gerontology. Series B, Psychological Sciences and Social Sciences 75, no. 7 (2020): 1515–1526. 30888038 10.1093/geronb/gbz037 PMC7424286

[4] Cabinet Office, Government of Japan , Annual Report on the Aging Society 2022 , Reiwa 4 edition (Cabinet Office, 2022).

[5] Consumer Affairs Agency (Japan) , Results of the FY2022 Basic Survey on Consumer Life (Reiwa 4 Fiscal Year—November 2022 Survey) (Consumer Affairs Agency, 2023), https://www.caa.go.jp/policies/policy/consumer_research/research_report/survey_002/assets/consumer_research_cms209_230831_01.pdf.

[6] S. A. Bass and F. G. Caro , “Productive Aging: A Conceptual Framework,” in Productive Aging: Concepts and Challenges, ed. N. Morrow‐Howell , J. Hinterlong , and M. Sherraden (Johns Hopkins University Press, 2001), 37–80.

[7] H. Murayama , M. Takase , S. Watanabe , K. Sugiura , I. Nakamoto , and Y. Fujiwara , “Employment in Old Age and All‐Cause Mortality: A Systematic Review,” Geriatrics & Gerontology International 22, no. 9 (2022): 705–714. 35924632 10.1111/ggi.14449

[8] S. Watanabe , H. Murayama , M. Takase , K. Sugiura , and Y. Fujiwara , “Longitudinal Association Between Work and Self‐Rated Health in Older Adults: A Systematic Review,” Nihon Koshu Eisei Zasshi. Japanese Journal of Public Health 69 (2022): 215–224. 34924494 10.11236/jph.21-060

[9] M. Takase , K. Sugiura , I. Nakamoto , S. Watanabe , and H. Murayama , “The Association Between Employment and Cognitive Function in Older Adults: A Systematic Review,” Geriatrics & Gerontology International 24 (2024): 1283–1291. 39557426 10.1111/ggi.15017

[10] Y. Fujiwara , S. Shinkai , E. Kobayashi , et al., “Engagement in Paid Work as a Protective Predictor of Basic Activities of Daily Living Disability in Japanese Urban and Rural Community‐Dwelling Elderly Residents: An 8‐Year Prospective Study,” Geriatrics & Gerontology International 16 (2016): 126–134. 25612931 10.1111/ggi.12441

[11] M. Ishizuka‐Inoue , A. Kawaguchi , S. Kashima , M. Nagai‐Tanima , and T. Aoyama , “Differences in Physical Activity and Mental Function According to the Employment Status of Elderly Japanese,” Journal of Occupational Health 65 (2023): e12411. 37347804 10.1002/1348-9585.12411 PMC10287044

[12] M. Tomida , Y. Nishita , C. Tange , et al., “Typology of Work–Family Balance Among Middle‐Aged and Older Japanese Adults,” Frontiers in Psychology 13 (2022): 751879. 35369186 10.3389/fpsyg.2022.751879 PMC8967286

[13] Cabinet Office, Government of Japan, Gender Equality Bureau , White Paper on Gender Equality (Cabinet Office, Government of Japan, 2014).

[14] Y. Fujiwara , S. Seino , Y. Nofuji , et al., “The Relationship Between Working Status in Old Age and Cause‐Specific Disability in Japanese Community‐Dwelling Older Adults With or Without Frailty: A 3.6‐Year Prospective Study,” Geriatrics & Gerontology International 23 (2023): 855–863. 37771279 10.1111/ggi.14686

[15] R. A. Karasek , “Job Demands, Job Decision Latitude, and Mental Strain: Implications for Job Redesign,” Administrative Science Quarterly 24 (1979): 285–308.

[16] A. B. Bakker and E. Demerouti , “Job Demands–Resources Theory: Taking Stock and Looking Forward,” Journal of Occupational Health Psychology 22 (2017): 273–285. 27732008 10.1037/ocp0000056

[17] A. Wrzesniewski and J. E. Dutton , “Crafting a Job: Revisioning Employees as Active Crafters of Their Work,” Academy of Management Review 26 (2001): 179–201.

[18] N. Maestas , “Back to Work: Expectations and Realizations of Work After Retirement,” Journal of Human Resources 45 (2010): 718–748. 24791018 10.1353/jhr.2010.0011 PMC4004604

[19] A. Börsch‐Supan , M. Brandt , C. Hunkler , et al., “Data Resource Profile: The Survey of Health, Ageing and Retirement in Europe (SHARE),” International Journal of Epidemiology 42 (2013): 992–1001. 23778574 10.1093/ije/dyt088 PMC3780997

[20] Y. Nemoto , T. Takahashi , K. Nonaka , et al., “Working for Only Financial Reasons Attenuates the Health Effects of Working Beyond Retirement Age: A 2‐Year Longitudinal Study,” Geriatrics & Gerontology International 20 (2020): 745–751. 32618090 10.1111/ggi.13941

[21] A. H. Maslow , “A Theory of Human Motivation,” Psychological Review 50 (1943): 370–396.

[22] H. Murayama , Y. Muto , M. Takase , I. Nakamoto , K. Nonaka , and Y. Fujiwara , “Effects of Employment Status and Motivations on the Onset of Social Isolation in Old Age: A 2.5‐Year Longitudinal Study,” JMA Journal 8 (2025): 1098–1107. 41220498 10.31662/jmaj.2025-0006 PMC12598144

[23] K. Mori , “Work Engagement Among Older Workers: A Systematic Review,” Journal of Occupational Health 66, no. 1 (2024): uiad008. 38258939 10.1093/joccuh/uiad008 PMC12516188

[24] T. Becker and S. T. Fiske , eds., “Workplace and Job Factors,” in Understanding the Aging Workforce (NCBI Bookshelf, 2022).

[25] P. V. AshaRani , D. Lai , J. Koh , and M. Subramaniam , “Purpose in Life in Older Adults: A Systematic Review on Its Associations With Physical Health, Cognitive Function, and Psychological Well‐Being,” International Journal of Environmental Research and Public Health 19 (2022): 5860. 35627396 10.3390/ijerph19105860 PMC9141815

[26] R. S. Weiss , S. A. Bass , H. K. Heimovitz , and M. Oka , “Japan's Silver Human Resource Centers and Participant Well‐Being,” Journal of Cross‐Cultural Gerontology 20 (2005): 47–66. 15870967 10.1007/s10823-005-3797-4

[27] U. Minami , H. Suzuki , M. Kuraoka , T. Koike , E. Kobayashi , and Y. Fujiwara , “Older Adults Looking for a Job Through Employment Support System in Tokyo,” PLoS One 11 (2016): e0159713. 27442115 10.1371/journal.pone.0159713 PMC4956197

[28] K. Morishita‐Suzuki , M. Nakamura‐Uehara , and T. Ishibashi , “The Improvement Effect of Working Through the Silver Human Resources Center on Pre‐Frailty Among Older People: A Two‐Year Follow‐Up Study,” BMC Geriatrics 23 (2023): 265. 37138219 10.1186/s12877-023-03978-z PMC10155134

[29] T. Abe , K. Fujita , T. Sagara , et al., “Associations Between Frailty Status, Work‐Related Accidents and Efforts for Safe Work Among Older Workers in Tokyo: A Cross‐Sectional Study,” Geriatrics & Gerontology International 23 (2023): 234–238. 36746782 10.1111/ggi.14557

[30] OECD , Promoting an Age‐Inclusive Workforce (OECD Publishing, 2020), https://www.oecd.org/en/publications/promoting‐an‐age‐inclusive‐workforce_59752153‐en.html/.

[31] World Health Organization , World Report on Ageing and Health (WHO, 2015), https://www.who.int/publications/i/item/9789241565042/.

[32] L. Bickerdike , A. Booth , P. M. Wilson , K. Farley , and K. Wright , “Social Prescribing: Less Rhetoric and More Reality. A Systematic Review of the Evidence,” BMJ Open 7 (2017): e013384. 10.1136/bmjopen-2016-013384 PMC5558801 28389486

[33] N. Ota , M. Ebihara , M. Aoki , et al., “Social Prescribing for Socially Isolated Older Adults in Rural Japan: A Qualitative Case Study,” Frontiers in Public Health 13 (2025): 1659713. 41179751 10.3389/fpubh.2025.1659713 PMC12571642

[34] Ministry of Health, Labor and Welfare (Japan) , Guidelines for the Community‐Based Integrated Long‐Term Care Prevention and Daily Life Support Program (Ministry of Health, Labor and Welfare, 2025), https://www.mhlw.go.jp/content/12300000/001285188.pdf.

